# Associations of paternal age with offspring under-five mortality and perinatal outcomes: a cohort study using claims data in Taiwan

**DOI:** 10.1136/bmjph-2024-001113

**Published:** 2024-11-28

**Authors:** Shi-Heng Wang, Jian-Te Lee, Mei-Chen Lin, Chi-Shin Wu, Wesley K Thompson, Chun-Chieh Fan

**Affiliations:** 1 National Center for Geriatrics and Welfare Research, National Health Research Institutes, Zhunan, Taiwan; 2 Department of Medical Research, China Medical University Hospital, China Medical University, Taichung, Taiwan; 3 Department of Pediatrics, National Taiwan University Hospital Yun-Lin Branch, Douliou, Taiwan; 4 Department of Psychiatry, National Taiwan University Hospital, Yunlin Branch, Douliu, Taiwan; 5 Center for Population Neuroscience and Genetics, Laureate Institute for Brain Research, Tulsa, Oklahoma, USA; 6 Department of Radiology, University of California San Diego, La Jolla, California, USA

**Keywords:** Epidemiology, Public Health, Age Factors

## Abstract

**Background:**

The causal relationship between advanced paternal age and offspring health is unclear, owing to familial confounders. This study examined the association of paternal age with offspring’s under-five mortality and perinatal outcomes, using sibling comparison analyses to account for familial confounding factors.

**Methods:**

A nationwide birth cohort study was designed based on Taiwan’s single-payer compulsory National Health Insurance programme. Individuals born between 2001 and 2015 were included, resulting in 2454 104 live-born singletons. Among them, 1513 222 individuals had full sibling(s) who were included in the sibling-comparison analyses. Logistic regression analyses were used to evaluate the main study cohort whereas conditional logistic regressions were used in the sibling-comparison analyses.

**Results:**

In the main cohort, paternal age categories showed a U-shaped relationship with offspring’s under-five mortality in the crude analysis, which attenuated towards the null hypothesis after accounting for the measured potential confounders. There was an increased risk of premature birth (gestational age <37 weeks), low birth weight (<2500 g), large for gestational age (90th percentile) and low 5 min Apgar Score (<7) in individuals with a paternal age of >35 years. Sibling-comparison analyses that accounted for unmeasured familial time-invariant confounders showed that younger siblings with older paternal age had a lower risk of under-five mortality, low birth weight, small for gestational age (10th percentile), congenital defects and low 5 min Apgar Score, and a higher risk of premature birth and large for gestational age.

**Conclusions:**

Children with older fathers had lower risks of under-five mortality, low birth weight, small for gestational age, congenital defects and low 5 min Apgar Score.

**WHAT IS ALREADY KNOWN ON THIS TOPIC**

- Paternal age has been shown to be associated with under-five mortality and adverse perinatal outcomes.
- Whether the association has a causal interpretation remains unclear.

**WHAT THIS STUDY ADDS**

- Using a nationwide birth cohort study of 2.45 million individuals in Taiwan, advanced paternal age was associated with an increased risk of premature birth, low birth weight, large for gestational age and low 5 min Apgar Score, but was not associated with offspring under five mortality in population-based analyses with adjustment for measured potential confounders.
- Among sibling pairs with the same familial predisposition, younger siblings with older paternal age had a higher risk of premature birth and large for gestational age and a lower risk of under-five mortality, low birth weight, small for gestational age, congenital defects and low 5 min Apgar Score.

**HOW THIS STUDY MIGHT AFFECT RESEARCH, PRACTICE OR POLICY**

- This study highlights the threat of unmeasured or residual familial confounding factors to causal inference for paternal age association and the importance of performing a family based study design.
- A more supportive familial environment provided by older fathers and a secular trend towards improving society counterbalance the negative consequences of reproductive ageing.

## Introduction

 Parental age is increasing in the developed world, and concerns regarding the potential adverse health consequences for offspring have been raised.^1^,^3^ Although the association between advanced maternal age and the risk of adverse health outcomes in offspring has been extensively studied, evidence for the health consequences of advanced paternal age is limited and lack diversity.^4^,^7^

Some population-based studies have shown that advanced paternal age is associated with under-five mortality^8^,^10^ and adverse perinatal outcomes.^11^,^15^ Higher de novo mutations in ageing men^316^,^24^ and age-related epigenetic modifications associated with the regulation of offspring gene expression^425^,^28^ have been implicated. Paternal imprinting during ageing has also been suggested to affect the placental epigenetic mechanisms.^29^

However, confounding factors associated with the selection of late fatherhood, such as genetic liability or psychosocial factors, may explain the paternal age association. A prior study suggested that the association of advanced paternal age with increased adult mortality could be substantially explained by early parental loss, which indicates that the mechanism behind the paternal age association may be psychosocial rather than physiological.^30^ Furthermore, sibling-comparison analyses that account for familial environmental factors and genetic predisposition found that advanced paternal age was associated with lower mortality, contrary to the findings from the population-based estimates without familial corrections.^31 32^ Older parents could provide a more supportive and well-resourced familial environment for their children than younger parents^33^,^35^ and, thus, could counterbalance the adverse effects of reproductive ageing. In addition, delayed childbearing also means that the offspring might live in a healthier and wealthier society.^31 32^

Whether confounding factors explain the association between advanced paternal age and offspring health remains unclear. Using a nationwide birth cohort study in Taiwan’s single-payer compulsory National Health Insurance programme, this study aimed to examine the association of paternal age with offspring’s under-five mortality and perinatal outcomes. Sibling comparison analyses were applied to further account for familial confounding factors and evaluate whether paternal age had an independent effect.

## Methods

### Study samples

A nationwide birth cohort study was conducted on individuals born between 1 January 2001 and 31 December 2015, who were followed up until 2020 in the National Health Insurance Research Database, which covers approximately 99% of Taiwan’s residents. We integrated several databases provided by the Health and Welfare Data Science Center, Ministry of Health and Welfare, Taiwan, all of which were linked using unique personal identifiers.

The Registry for Beneficiaries and Maternal and Child Health databases were used to ascertain kinship. The procedure has been validated with high accuracy (>98%) and detailed in a prior publication.^2^ Full siblings were defined as two individuals with the same parents. Twins are defined as individuals born on the same day and from the same parents. The Birth Certificate Application database was used to retrieve the detailed health conditions of newborns and their labour characteristics. Inpatient and outpatient claims databases were used to identify neuropsychiatric diagnoses. The Registry for Beneficiaries database was used to retrieve the insurance amount and urbanisation level of residents. The Death Certification database was used to determine the vital statuses of the study population.

A total of 2727 000 eligible individuals born between 2001 and 2015 with information on both parent and labour characteristics were retrieved. Twins, triplets or high-order multiples (n=134 134); stillbirths (n=33); singletons born at a gestational age <32 weeks or >42 weeks (n=986); newborns with foreign mothers (n=135 493); those with missing insurance levels (n=1400) and urbanisation data (n=850) were excluded from the study. Finally, 2454 104 live-born singletons from 1652 518 families were included in the cohort, and 1513 222 individuals with full siblings from 711 636 families were selected as the sibling-comparison cohort.

### Study outcomes

The outcomes of interest were under-five mortality, under-one mortality and perinatal outcomes. The perinatal outcomes evaluated were premature birth (gestational age <37 weeks), low birth weight (<2500 g), congenital defects, low 5 min Apgar Score (<7), small for gestational age (SGA, 10th percentile), and large for gestational age (LGA, 90th percentile). All of the variables were categorised as dichotomous.

### Paternal age and covariates

The paternal age was calculated by subtracting the birth date of the offspring from that of the father. Paternal age was categorised as <20 years, 20–24 years, 25–29 years, 30–34 years, 35–39 years, 40–44 years, 45–49 years and ≥50 years of age.

Several covariates were considered, including sex, birth year, maternal age (coded as a categorical variable, classified into the following groups: <20 years, 20–24 years, 25–29 years, 30–34 years, 35–39 years and ≥40 years, as well as a continuous variable), parity, delivery methods (classified into the following groups: unassisted vaginal birth, assisted vaginal birth and caesarean section),^36 37^ urbanisation level (classified into the following groups: urban, suburban and rural), and insurance level. The urbanisation level classification of a residential district was based on population density, proportion of residents with higher education, older and agricultural population, and the number of physicians per 100 000 people.^38^ The payroll-related insurance amount was used as a proxy measure of the family’s economic status and was categorised into three levels based on tertiles.

### Statistical analysis

We estimated the ORs and 95% CIs for paternal age associations using logistic regression models. The first model was a crude model without adjustments. The second model was adjusted for maternal age, offspring sex, parity, delivery method and calendar year of birth. The fully adjusted model was further adjusted for the family insurance amount and the urbanisation level of the residential area.

To better consider residual confounding factors or unmeasured confounders shared within a family, we performed sibling-comparison analysis. Sibling pairs that were discordant in the outcome of interest were selected to construct conditional logistic regression models.^39^ The sample size of the sibling-comparison cohort is summarised in online supplemental table 1. The effects of paternal and maternal age could not be separated because they were perfectly collinear within the families. The range of paternal age within a family was small; hence, we analysed paternal age as a continuous variable rather than a categorical variable, as described above. Furthermore, to consider parents’ advancing age in the sibling-comparison analyses, we used parity as a proxy for father’s advancing age. The OR for perinatal outcomes given parity, which reflects both paternal and maternal age, was estimated after adjusting for offspring sex and delivery method. If advanced paternal age played a causal role in the aetiology of perinatal outcomes and under-five and under-one mortality, younger offspring with higher parity would be expected to have an increased risk of these outcomes. Offspring parity was categorised as first-born, second-born, third-born or later-born.

Subgroup analyses stratified by categorial maternal age (<24 years, 25–29 years, 30–34 years and ≥35 years) were performed in both the population and the sibling-comparison analyses.

All statistical analyses were performed using SAS statistical package (V.9.4; SAS Institute, Cary, North Carolina, USA).

## Results

### Baseline characteristics

Among the 2454 104 individuals in the population cohort, we identified 7114 (0.3%) with under-five mortality, 4830 (0.2%) with under-one mortality, 172 625 (7.0%) with premature birth, 147 522 (6.0%) with low birth weight, 11 917 (0.5%) with congenital defect, 9156 (0.4%) with low 5 min Apgar Score, 235 289 (9.6%) with SGA and 244 632 (10.0%) with LGA. The distribution of participant demographics, family characteristics, under-five and under-one mortality, and perinatal outcomes in the population cohort and sibling-comparison cohort are shown in table 1 (a detailed distribution stratified by paternal age group is shown in online supplemental table 2).

**Table 1:** Distribution of demographics, family characteristics, maternal and perinatal outcomes in the population cohort (n=2454 104) and sibling-comparison cohort (n=1513 222)

Variable	Population cohort(n=2454 104)n (%)	Sibling-comparison cohort(n=1513 222)n (%)
Sex		
Female	1174 743 (47.9)	738 520 (48.8)
Male	1 279 361 (52.1)	774 702 (51.2)
Birth year		
2001–2005	766 379 (31.2)	417 809 (27.6)
2006–2010	802 883 (32.7)	578 736 (38.2)
2011–2015	884 842 (36.1)	516 677 (34.1)
Insurance level		
<26 400	804 033 (32.8)	430 758 (28.5)
26 400–45800	813 557 (33.2)	502 210 (33.2)
>45 800	836 514 (34.1)	580 254 (38.3)
Urbanisation		
Urban	1359 143 (55.4)	811 408 (53.6)
Suburban	897 314 (36.6)	570 796 (37.7)
Rural	197 647 (8.1)	131 018 (8.7)
Paternal age, years		
<20	11 935 (0.5)	5755 (0.4)
20–24	146 484 (6.0)	94 518 (6.3)
25–29	589 202 (24.0)	397 530 (26.3)
30–34	950 863 (38.7)	599 833 (39.6)
35–39	554 697 (22.6)	322 181 (21.3)
40–44	160 230 (6.5)	78 706 (5.2)
45–49	32 393 (1.3)	12 422 (0.8)
≥50	8300 (0.3)	2277 (0.2)
Maternal age, years		
<20	50 671 (2.1)	27 238 (1.8)
20–24	323 936 (13.2)	202 772 (13.4)
25–29	817 172 (33.3)	517 522 (34.2)
30–34	888 867 (36.2)	549 300 (36.3)
35–39	327 823 (13.4)	195 206 (12.9)
≥40	45 635 (1.9)	21 184 (1.4)
Parity		
1	1622 650 (66.1)	712 042 (47.1)
2	725 423 (29.6)	712 042 (47.1)
≥3	106 031 (4.3)	89 138 (5.9)
Delivery method		
Unassisted vaginal birth	1409 449 (57.4)	908 007 (60.0)
Vaginal birth assisted by forceps or vacuum extraction	205 228 (8.4)	119 497 (7.9)
Caesarean section	839 427 (34.2)	485 718 (32.1)
Perinatal outcome		
Under-five mortality	7114 (0.3)	5472 (0.4)
Under-one mortality	4830 (0.2)	3907 (0.3)
Premature birth	172 625 (7.0)	101 047 (6.7)
Low birth weight	147 522 (6.0)	83 856 (5.5)
Congenital defect	11 917 (0.5)	6950 (0.5)
Low 5 min Apgar Score	9156 (0.4)	5155 (0.3)
SGA	235 289 (9.6)	141 113 (9.3)
LGA	244 632 (10.0)	145 455 (9.6)

LGA, large for gestational ageSGA, small for gestational age

### Under-five mortality and under-one mortality

**Figure 1:**
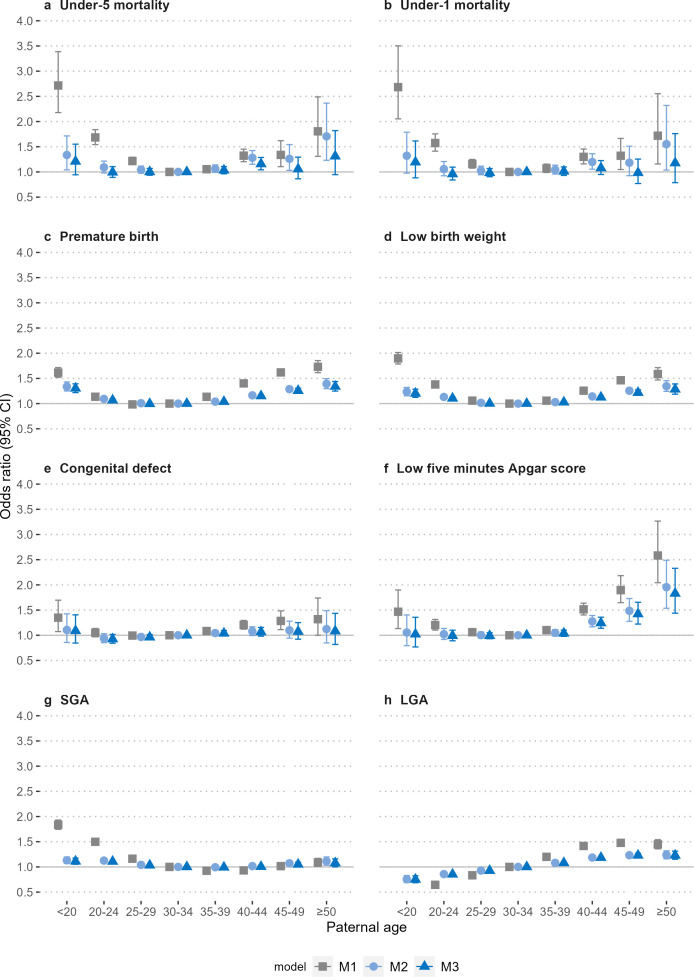
Association of paternal age with the risk of perinatal outcomes. OR and 95% CI. Model 1 was a crude estimate without adjustment. Model 2 adjusted for maternal age, offspring’s sex, parity, delivery method and calendar year of birth. Model 3 further adjusted the family’s insurance amount and the urbanisation level of the residential area. LGA, large for gestational age; SGA, small for gestational age.

In the population analyses, the estimated association between paternal age and risk of perinatal outcomes in Models 1–3 is shown in figure 1 (detailed numbers in online supplemental table 3). In the crude model, paternal age categories had a U-shaped relationship with the offspring’s under-five and under-one mortality (figure 1). After adjusting for categorical maternal age, offspring sex, parity, delivery method, calendar year of birth, family insurance and resident urbanisation in the fully adjusted model, the associations with paternal age were attenuated towards null. Adjusting for continuous maternal age produced slightly different estimates of paternal age associations (online supplemental table 4). Maternal age categories also had a U-shaped relationship with the offspring’s under-five and under-one mortality in the crude model, and the associations were attenuated towards the null in the fully adjusted model (online supplemental figure 1; detailed numbers in online supplemental table 5).

**Figure 2:**
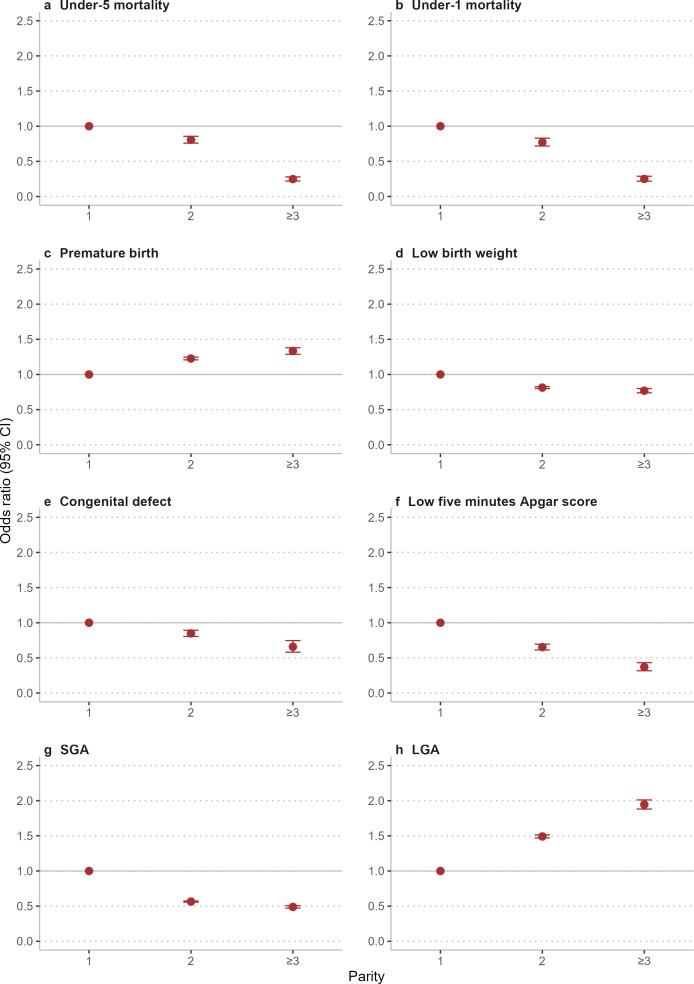
Association of parity with the risk of perinatal outcomes in the sibling-comparison analyses. OR (error bar is 95% CI) with adjustment for offspring’s sex and delivery method. LGA, large for gestational age; SGA, small for gestational age.

The results of the association between paternal age or parity and under-five and under-one mortality in the sibling-comparison analysis are presented in online supplemental table 6. The OR for per-year increase in paternal/maternal age was 0.87 (95% CI 0.86 to 0.89) and 0.88 (95% CI 0.87 to 0.90) in under-five and under-one mortality, respectively. Among the sib pairs with the same familial predisposition, younger siblings with higher parity had a lower risk of under-five and under-one mortality (figure 2).

### Perinatal outcomes

In both the crude and fully adjusted models (figure 1), paternal age categories had a U-shaped relationship with offspring’s premature birth and low birth weight; paternal age <30 years was associated with offspring SGA, and paternal age was linearly associated with offspring LGA. Paternal age categories had a U-shaped relationship with congenital defects and low 5 min Apgar Score; however, after full adjustment, the association for congenital defects attenuated towards the null, and paternal age >35 years was still associated with a higher risk of low 5 min Apgar Score in the offspring. In general, maternal and paternal age categories presented a similar pattern of association with perinatal outcomes (online supplemental figure 1).

The sibling-comparison analyses revealed that the OR for per-year increase in paternal/maternal age was 1.05 (95% CI 1.04 to 1.05) for premature birth, 0.97 (95% CI 0.96 to 0.97) for low birth weight, 0.94 (95% CI 0.93 to 0.95) for congenital defect, 0.89 (95% CI 0.87 to 0.90) for low 5 min Apgar Score, 0.89 (95% CI 0.89 to 0.90) for SGA and 1.10 (95% CI 1.10 to 1.10) for LGA (online supplemental table 6). Younger siblings with higher parity had higher risks of premature birth and LGA and lower risks of low birth weight, SGA, congenital defects and low 5 min Apgar Score (figure 2).

### Stratified analyses

The estimated associations between paternal age and the risk of perinatal outcomes stratified by categorical maternal age in the fully adjusted model are shown in online supplemental table 7. Stratifying the total population into different maternal age groups produced relatively imprecise estimates of the paternal age associations. The maternal age-stratified results for the sibling-comparison analyses are shown in online supplemental table 8. Generally, the association between paternal age or parity and perinatal outcomes was similar across maternal age groups.

## Discussion

This nationwide birth cohort study of more than 2.45 million Taiwanese children assessed the impact of paternal age on offspring mortality and perinatal outcomes. Population analyses suggested that paternal age was not associated with offspring under-five and under-one mortality; however, the sibling analyses suggest that younger siblings have a lower mortality risk within the same family. For perinatal outcomes, among sib pairs with the same familial predisposition, younger siblings had higher risks of premature birth and LGA and lower risks of low birth weight, SGA, congenital defects and low 5 min Apgar Score.

Children born to older fathers have been shown to be at a higher risk of under-five mortality in a Danish population-based study and in a pooled analysis in low-income and middle-income countries;^8 9^ however, these findings may be confounded by shared familial factors or secular trends. Two Swedish studies with 1.9 million and 5.2 million births between 1938 and 1960,^31^ and 1932 and 1987,^32^ respectively, suggested that the association between advanced paternal age and offspring adult mortality may be explained by secular increases in longevity during the extended study period. This study explored the independent role of advanced paternal age in offspring under-five mortality among 2.45 million Taiwanese births from 2001 to 2015, which is a relatively short period in modern society. In the main cohort, we observed that both paternal and maternal age categories had a U-shaped relationship with the offspring’s under-five and under-one mortality and that both paternal age >35 years and maternal age >35 years were associated with a higher risk of offspring under-five and under-one mortality in the univariate analysis. However, these associations were attenuated towards the null hypothesis after adjusting for potential confounding factors. Notably, adjusting for categorical and continuous maternal age produced slightly different estimates of paternal age associations. Furthermore, with our sibling comparison analyses that removed invariant family characteristics, younger siblings with older parental age had a lower risk of under-five and under-one mortality. Similar trends were also found in the maternal age-stratified analyses. Taken together, the observed negative consequences of reproductive ageing for under-five mortality may be counterbalanced by a more supportive and well-resourced familial environment provided by older parents as well as by a secular trend of improving society.

Previous evidence of the association between advanced paternal age and adverse perinatal outcomes has been inconsistent. A study with 1.5 million births in Italy observed that advanced paternal age was associated with offspring preterm birth;^14^ however, this association was not observed in another study with 1 million births in Ohio.^40^ A study with 2.6 million births in the USA found higher risks for preterm birth, low birth weight, SGA and low Apgar Score among children born to fathers <20 years of age, but not fathers >30 years.^41^ A large-scale study with over 40 million individuals born between 2007 and 2016 in the USA showed that children born to fathers of advanced paternal age have an increased risk of premature birth, low birth weight and low Apgar Score,^11^ and these findings were replicated in a study with 17 million individuals born between 2016 and 2021 in the USA.^15^ Simultaneously, our population-based analyses in Taiwan of East Asian populations observed that paternal age >35 years was associated with higher risks of premature birth, low birth weight, LGA and low 5 min Apgar Score. The associations for premature birth and LGA were replicated in our sibling-comparison analyses. However, younger siblings with advanced parental age had lower risks of low birth weight, SGA, congenital defects and low 5 min Apgar Score among sib pairs with the same familial predisposition, suggesting the confounding roles of familial factors and secular improvements in medical technology, such as prenatal examination.

The population-based analyses demonstrated an increased risk of mortality and several perinatal outcomes in children born to very young fathers, which is consistent with the results of previous studies.^8 11 15 30 32 41^ Early parenthood interferes with education and employment,^42^,^44^ which leads to adverse familial economics, unsupportive home environments, adverse parenting behaviours, and subsequent adverse perinatal outcomes and offspring mortality.

This study has several limitations that should be noted when interpreting our findings. First, information on parental education was not available, and we adjusted for the family insurance amount as a proxy for the family’s socioeconomic status in the population-based analyses. Our sibling comparison analyses eliminated unmeasured familial time-invariant confounders. Second, within-family sibling comparisons cannot explicitly differentiate the paternal age effect from the maternal age effect, because paternal age and maternal age have the same change from one sibling to the next within a family. Third, compared with the total cohort, the sample size is much reduced in the sibling-comparison cohort (table 1); thus, selection bias is possible. The post-traumatic experience from the previous child might influence the willingness for bearing subsequent children; therefore, our sibling-comparison analyses may have recruited a healthier proportion of the total study population. Finally, our sibling-comparison analyses may be biased due to carryover effects.^45^ The family already having an older child with adverse perinatal outcomes may lead to paying more attention in preparing for and during subsequent pregnancies. Time-varying and non-shared family confounding factors were not considered in sibling comparison analyses. Family socioeconomic status and parenting behaviours may improve over time between older and younger children, and the association of paternal age with offspring’s risk of mortality and perinatal outcomes may be biased towards the null hypothesis.

## Conclusions

This nationwide birth cohort study with population and sibling analyses suggests that children with older fathers have a lower risk of under-five and under-one mortality. Although children with older fathers have an increased risk of premature birth, they have a lower risk of low birth weight, SGA, congenital defects and low 5 min Apgar Score. Our study highlights the threat of unmeasured or residual familial confounding factors to causal inference for paternal age association and the importance of performing a family based study design.

## supplementary material

10.1136/bmjph-2024-001113 online supplemental file 1

## Data Availability

All data relevant to the study are included in the article or uploaded as supplementary information.
