# Supplementary material for: Associations of paternal age with offspring under-five mortality and perinatal outcomes: a cohort study using claims data in Taiwan
Source: BMJ Public Health. 2024 Nov 28;2(2):e001113. doi: 10.1136/bmjph-2024-001113 (PMC11816320; doi:10.1136/bmjph-2024-001113)
Supplement: online supplemental file 1 [file bmjph-2-2-s001.pdf]

Supplementary Online Content

Supplementary Figure 1. Association of maternal age with the risk of perinatal outcomes. Odds ratio and 95% confidence interval. Model 1 was crude estimate without adjustment. Model 2 adjusted for paternal age, offspring' sex, parity, delivery method, and calendar year of birth. Model 3 further adjusted the family's insurance amount and the urbanization level of the residential area.

Supplementary Table 1. Sample size kept for sibling-comparison analyses

Supplementary Table 2. Demographic, family characteristics, maternal and perinatal outcomes by paternal age group

Supplementary Table 3. Association of paternal age with the risk of perinatal outcomes (adjustment for categorical maternal age)

Supplementary Table 4. Association of paternal age with the risk of perinatal outcomes (adjustment for continuous maternal age)

Supplementary Table 5. Association of maternal age with the risk of perinatal outcomes

Supplementary Table 6. Association between parity and perinatal outcomes in the sibling-comparison analyses

Supplementary Table 7. Association of paternal age with the risk of perinatal outcomes stratified by categorical maternal age

Supplementary Table 8. Association between parity and perinatal outcomes stratified by categorical maternal age in the sibling-comparison analyses

Supplementary Figure 1. Association of maternal age with the risk of perinatal outcomes. Odds ratio and 95% confidence interval. Model 1 was crude estimate without adjustment. Model 2 adjusted for paternal age, offspring' sex, parity, delivery method, and calendar year of birth. Model 3 further adjusted the family's insurance amount and the urbanization level of the residential area.

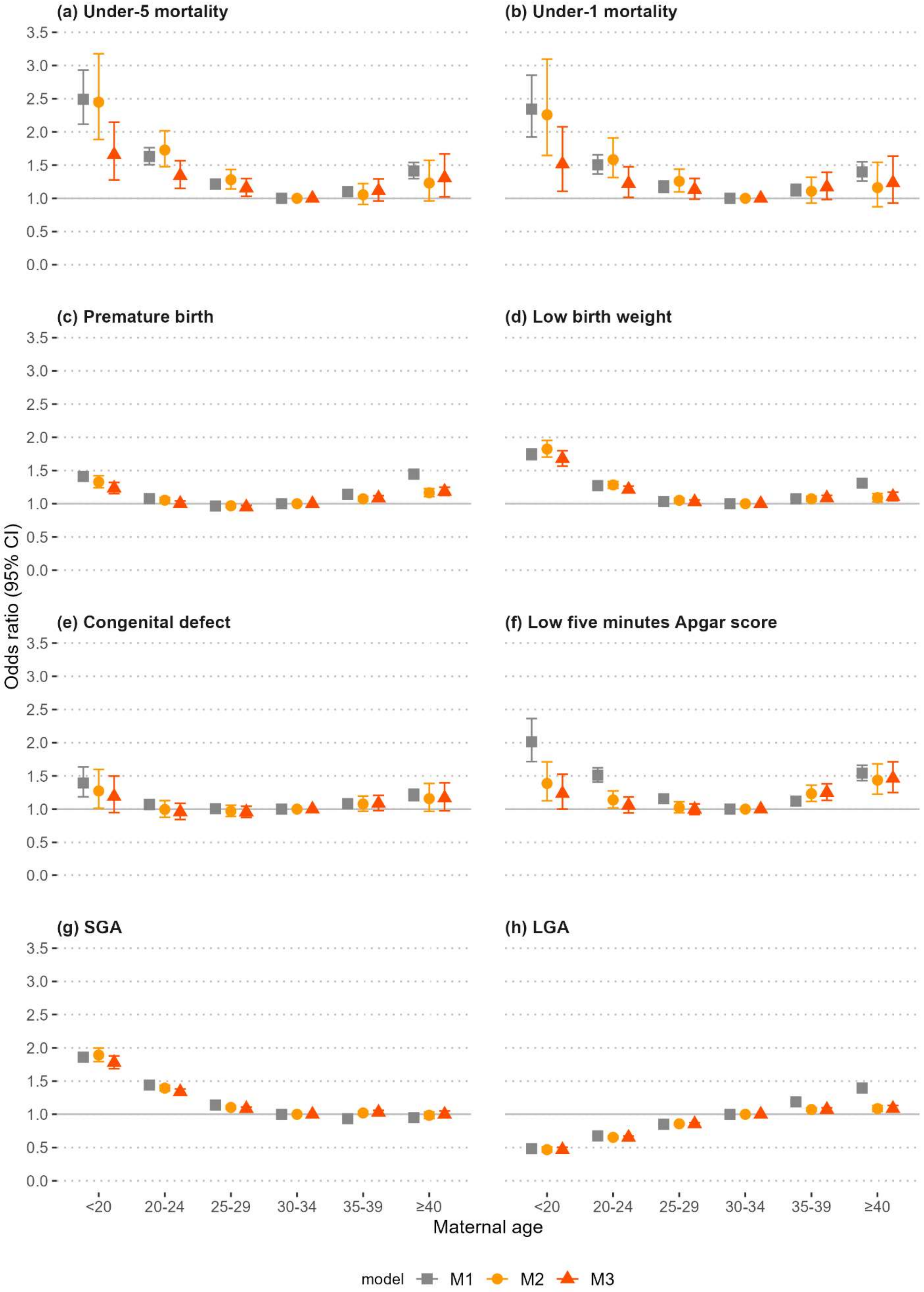

Supplementary Table 1. Sample size kept for sibling-comparison analyses

Perinatal outcome	N
Under-5 mortality	12,889
Under-1 mortality	9,019
Premature birth	172,464
Low birth weight	144,263
Congenital defect	15,005
Low five minutes Apgar score	12,282
Small for gestational age (SGA)	219,129
Large for gestational age (LGA)	219,198

Supplementary Table 2. Demographic, family characteristics, maternal and perinatal outcomes by paternal age group

	Paternal age								
	<20 (N=11935)	20-24 (N=146484)	25-29 (N=589202)	30-34 (N=950863)	35-39 (N=554697)	40-44 (N=160230)	45-49 (N=32393)	≥50 (N=8300)	Total (N=2454104)
	n (%)	n (%)	n (%)	n (%)	n (%)	n (%)	n (%)	n (%)	n (%)
Sex									
Female	5720 (47.9)	70501 (48.1)	282725 (48.0)	455543 (47.9)	264672 (47.7)	76208 (47.6)	15466 (47.7)	3908 (47.1)	1174743 (47.9)
Male	6215 (52.1)	75983 (51.9)	306477 (52.0)	495320 (52.1)	290025 (52.3)	84022 (52.4)	16927 (52.3)	4392 (52.9)	1279361 (52.1)
Birth year									
2001-2005	5388 (45.1)	66788 (45.6)	226648 (38.5)	279045 (29.3)	141313 (25.5)	37877 (23.6)	7342 (22.7)	1978 (23.8)	766379 (31.2)
2006-2010	3447 (28.9)	43631 (29.8)	200906 (34.1)	314722 (33.1)	176910 (31.9)	50887 (31.8)	9922 (30.6)	2458 (29.6)	802883 (32.7)
2011-2015	3100 (26.0)	36065 (24.6)	161648 (27.4)	357096 (37.6)	236474 (42.6)	71466 (44.6)	15129 (46.7)	3864 (46.6)	884842 (36.1)
Insurance level									
<26400	6999 (58.6)	73700 (50.3)	216043 (36.7)	272038 (28.6)	162566 (29.3)	55322 (34.5)	13202 (40.8)	4163 (50.2)	804033 (32.8)
26400-45800	4103 (34.4)	54901 (37.5)	221465 (37.6)	311330 (32.7)	164013 (29.6)	46019 (28.7)	9472 (29.2)	2254 (27.2)	813557 (33.2)
>45800	833 (7.0)	17883 (12.2)	151694 (25.7)	367495 (38.6)	228118 (41.1)	58889 (36.8)	9719 (30.0)	1883 (22.7)	836514 (34.1)
Urbanization									
Urban	3920 (32.8)	53793 (36.7)	276859 (47.0)	549818 (57.8)	347972 (62.7)	101629 (63.4)	20125 (62.1)	5027 (60.6)	1359143 (55.4)
Sub-urban	5682 (47.6)	69247 (47.3)	251585 (42.7)	336745 (35.4)	174008 (31.4)	47979 (29.9)	9605 (29.7)	2463 (29.7)	897314 (36.6)
Rural	2333 (19.5)	23444 (16.0)	60758 (10.3)	64300 (6.8)	32717 (5.9)	10622 (6.6)	2663 (8.2)	810 (9.8)	197647 (8.1)
Maternal age									
<20	9192 (77.0)	23883 (16.3)	12065 (2.0)	3262 (0.3)	1178 (0.2)	620 (0.4)	310 (1.0)	161 (1.9)	50671 (2.1)
20-24	2527 (21.2)	100421 (68.6)	149801 (25.4)	54502 (5.7)	12687 (2.3)	2973 (1.9)	757 (2.3)	268 (3.2)	323936 (13.2)
25-29	163 (1.4)	19647 (13.4)	360608 (61.2)	343002 (36.1)	78036 (14.1)	12552 (7.8)	2413 (7.4)	751 (9.0)	817172 (33.3)
30-34	44 (0.4)	2175 (1.5)	60968 (10.3)	500220 (52.6)	272541 (49.1)	43988 (27.5)	7022 (21.7)	1909 (23.0)	888867 (36.2)
35-39	6 (0.1)	319 (0.2)	5371 (0.9)	47570 (5.0)	181809 (32.8)	77126 (48.1)	12678 (39.1)	2944 (35.5)	327823 (13.4)
≥40	3 (0.0)	39 (0.0)	389 (0.1)	2307 (0.2)	8446 (1.5)	22971 (14.3)	9213 (28.4)	2267 (27.3)	45635 (1.9)
Parity									

1	10917 (91.5)	116351 (79.4)	432474 (73.4)	620941 (65.3)	319349 (57.6)	95075 (59.3)	21485 (66.3)	6058 (73.0)	1622650 (66.1)
2	954 (8.0)	27076 (18.5)	139377 (23.7)	292571 (30.8)	201842 (36.4)	53195 (33.2)	8662 (26.7)	1746 (21.0)	725423 (29.6)
≥3	64 (0.5)	3057 (2.1)	17351 (2.9)	37351 (3.9)	33506 (6.0)	11960 (7.5)	2246 (6.9)	496 (6.0)	106031 (4.3)
Delivery method									
Unassisted vaginal birth	8291 (69.5)	95360 (65.1)	357292 (60.6)	549714 (57.8)	300309 (54.1)	79382 (49.5)	15237 (47.0)	3864 (46.6)	1409449 (57.4)
Vaginal birth assisted by forceps or vacuum extraction	1411 (11.8)	14371 (9.8)	55440 (9.4)	81389 (8.6)	39815 (7.2)	10309 (6.4)	1959 (6.0)	534 (6.4)	205228 (8.4)
Caesarian section	2233 (18.7)	36753 (25.1)	176470 (30.0)	319760 (33.6)	214573 (38.7)	70539 (44.0)	15197 (46.9)	3902 (47.0)	839427 (34.2)
Perinatal outcome									
Under-five mortality	82 (0.7)	626 (0.4)	1819 (0.3)	2416 (0.3)	1485 (0.3)	538 (0.3)	110 (0.3)	38 (0.5)	7114 (0.3)
Under-one mortality	56 (0.5)	404 (0.3)	1197 (0.2)	1668 (0.2)	1040 (0.2)	365 (0.2)	75 (0.2)	25 (0.3)	4830 (0.2)
Premature birth	1220 (10.2)	10860 (7.4)	38216 (6.5)	62610 (6.6)	41095 (7.4)	14404 (9.0)	3318 (10.2)	902 (10.9)	172625 (7.0)
Low birth weight	1207 (10.1)	11080 (7.6)	34836 (5.9)	53243 (5.6)	32746 (5.9)	11109 (6.9)	2587 (8.0)	714 (8.6)	147522 (6.0)
Congenital defect	75 (0.6)	717 (0.5)	2735 (0.5)	4439 (0.5)	2806 (0.5)	900 (0.6)	194 (0.6)	51 (0.6)	11917 (0.5)
Low 5-minute Apgar score	59 (0.5)	596 (0.4)	2116 (0.4)	3218 (0.3)	2068 (0.4)	820 (0.5)	207 (0.6)	72 (0.9)	9156 (0.4)
SGA	1868 (15.7)	19264 (13.2)	61941 (10.5)	87258 (9.2)	47351 (8.5)	13770 (8.6)	3015 (9.3)	822 (9.9)	235289 (9.6)
LGA	557 (4.7)	9644 (6.6)	49248 (8.4)	93724 (9.9)	64336 (11.6)	21482 (13.4)	4507 (13.9)	1134 (13.7)	244632 (10.0)

SGA: small for gestational age; LGA: large for gestational age

Supplementary Table 3. Association of paternal age with the risk of perinatal outcomes (adjustment for categorical maternal age)

	Logistic regression											
	Model 1				Model 2				Model 3			
	OR	LCL	UCL	p-value	OR	LCL	UCL	p-value	OR	LCL	UCL	p-value
Under-5 mortality												
Paternal age												
<20	2.72	2.18	3.39	<.0001	1.34	1.04	1.72	0.0229	1.21	0.94	1.55	0.1342
20-24	1.69	1.54	1.84	<.0001	1.09	0.98	1.22	0.1125	0.99	0.89	1.11	0.8914
25-29	1.22	1.14	1.29	<.0001	1.04	0.97	1.12	0.2254	1.00	0.93	1.07	0.9890
30-34	1.00	-	-	-	1.00	-	-	-	1.00	-	-	-
35-39	1.05	0.99	1.12	0.1126	1.06	0.99	1.14	0.0889	1.03	0.96	1.11	0.3641
40-44	1.32	1.20	1.45	<.0001	1.28	1.15	1.42	<.0001	1.16	1.04	1.29	0.0063
45-49	1.34	1.11	1.62	0.0029	1.26	1.03	1.54	0.0249	1.06	0.86	1.29	0.5911
>=50	1.81	1.31	2.49	0.0003	1.71	1.23	2.37	0.0014	1.31	0.95	1.82	0.1036
Under-1 mortality												
Paternal age												
<20	2.68	2.05	3.50	<.0001	1.32	0.98	1.79	0.0717	1.19	0.88	1.62	0.2488
20-24	1.57	1.41	1.76	<.0001	1.06	0.92	1.21	0.4224	0.96	0.84	1.10	0.5389
25-29	1.16	1.08	1.25	0.0001	1.03	0.94	1.11	0.5526	0.98	0.91	1.07	0.6767
30-34	1.00	-	-	-	1.00	-	-	-	1.00	-	-	-
35-39	1.07	0.99	1.16	0.0918	1.04	0.96	1.13	0.3253	1.01	0.93	1.10	0.7872
40-44	1.30	1.16	1.46	<.0001	1.20	1.05	1.36	0.0057	1.08	0.95	1.23	0.2490
45-49	1.32	1.05	1.67	0.0186	1.18	0.93	1.51	0.1790	0.98	0.77	1.26	0.8920
>=50	1.72	1.16	2.55	0.0072	1.55	1.04	2.32	0.0325	1.18	0.79	1.76	0.4284
Premature birth												
Paternal age												
<20	1.62	1.52	1.72	<.0001	1.34	1.25	1.43	<.0001	1.30	1.22	1.39	<.0001
20-24	1.14	1.11	1.16	<.0001	1.09	1.07	1.12	<.0001	1.07	1.04	1.10	<.0001

25-29	0.98	0.97	1.00	0.0163	1.01	0.99	1.02	0.2704	1.00	0.98	1.01	0.5797
30-34	1.00	-	-	-	1.00	-	-	-	1.00	-	-	-
35-39	1.14	1.12	1.15	<.0001	1.04	1.03	1.05	<.0001	1.04	1.02	1.05	<.0001
40-44	1.40	1.38	1.43	<.0001	1.16	1.14	1.19	<.0001	1.15	1.13	1.18	<.0001
45-49	1.62	1.56	1.68	<.0001	1.29	1.24	1.34	<.0001	1.26	1.21	1.31	<.0001
>=50	1.73	1.61	1.85	<.0001	1.39	1.30	1.50	<.0001	1.34	1.25	1.44	<.0001
Low birth weight												
Paternal age												
<20	1.90	1.79	2.01	<.0001	1.24	1.16	1.32	<.0001	1.20	1.12	1.29	<.0001
20-24	1.38	1.35	1.41	<.0001	1.13	1.10	1.16	<.0001	1.10	1.08	1.13	<.0001
25-29	1.06	1.05	1.07	<.0001	1.02	1.00	1.03	0.0510	1.00	0.99	1.02	0.6674
30-34	1.00	-	-	-	1.00	-	-	-	1.00	-	-	-
35-39	1.06	1.04	1.07	<.0001	1.03	1.01	1.05	0.0002	1.03	1.01	1.04	0.0007
40-44	1.26	1.23	1.28	<.0001	1.14	1.12	1.17	<.0001	1.13	1.10	1.15	<.0001
45-49	1.46	1.40	1.53	<.0001	1.26	1.20	1.31	<.0001	1.22	1.17	1.27	<.0001
>=50	1.59	1.47	1.71	<.0001	1.35	1.24	1.46	<.0001	1.28	1.19	1.39	<.0001
Congenital defects												
Paternal age												
<20	1.35	1.07	1.70	0.0103	1.11	0.86	1.43	0.4387	1.09	0.85	1.41	0.5103
20-24	1.05	0.97	1.14	0.2384	0.94	0.85	1.03	0.1836	0.93	0.84	1.02	0.1055
25-29	0.99	0.95	1.04	0.8143	0.97	0.92	1.02	0.1981	0.96	0.91	1.01	0.1468
30-34	1.00	-	-	-	1.00	-	-	-	1.00	-	-	-
35-39	1.08	1.03	1.14	0.0008	1.04	0.99	1.10	0.1104	1.04	0.99	1.09	0.1466
40-44	1.20	1.12	1.29	<.0001	1.08	0.99	1.17	0.0690	1.07	0.98	1.16	0.1297
45-49	1.29	1.11	1.48	0.0007	1.10	0.94	1.28	0.2273	1.07	0.92	1.25	0.3608
>=50	1.32	1.00	1.74	0.0501	1.12	0.85	1.49	0.4201	1.08	0.82	1.44	0.5767
Low five minutes Apgar score												
Paternal age												

<20	1.47	1.13	1.90	0.0036	1.06	0.79	1.40	0.7127	1.02	0.77	1.36	0.8876
20-24	1.20	1.10	1.31	<.0001	1.02	0.92	1.14	0.7002	0.99	0.89	1.10	0.8482
25-29	1.06	1.01	1.12	0.0335	1.00	0.94	1.07	0.9270	0.99	0.93	1.05	0.7425
30-34	1.00	-	-	-	1.00	-	-	-	1.00	-	-	-
35-39	1.10	1.04	1.17	0.0006	1.05	0.99	1.11	0.1353	1.04	0.98	1.10	0.2090
40-44	1.52	1.40	1.64	<.0001	1.28	1.17	1.39	<.0001	1.24	1.14	1.36	<.0001
45-49	1.90	1.65	2.18	<.0001	1.49	1.28	1.73	<.0001	1.42	1.22	1.66	<.0001
>=50	2.58	2.04	3.27	<.0001	1.96	1.54	2.49	<.0001	1.83	1.44	2.33	<.0001
SGA												
Paternal age												
<20	1.84	1.75	1.93	<.0001	1.13	1.07	1.20	<.0001	1.11	1.05	1.18	0.0002
20-24	1.50	1.47	1.52	<.0001	1.13	1.10	1.15	<.0001	1.11	1.09	1.13	<.0001
25-29	1.16	1.15	1.18	<.0001	1.04	1.03	1.05	<.0001	1.03	1.02	1.05	<.0001
30-34	1.00	-	-	-	1.00	-	-	-	1.00	-	-	-
35-39	0.92	0.91	0.94	<.0001	0.99	0.98	1.01	0.3214	0.99	0.98	1.00	0.1977
40-44	0.93	0.91	0.95	<.0001	1.02	1.00	1.04	0.1348	1.01	0.98	1.03	0.6254
45-49	1.02	0.98	1.06	0.4225	1.07	1.03	1.12	0.0007	1.05	1.01	1.09	0.0172
>=50	1.09	1.01	1.17	0.0225	1.11	1.03	1.20	0.0046	1.08	1.00	1.16	0.0457
LGA												
Paternal age												
<20	0.45	0.41	0.49	<.0001	0.76	0.69	0.83	<.0001	0.75	0.69	0.82	<.0001
20-24	0.65	0.63	0.66	<.0001	0.86	0.84	0.88	<.0001	0.85	0.83	0.88	<.0001
25-29	0.83	0.83	0.84	<.0001	0.93	0.92	0.94	<.0001	0.93	0.91	0.94	<.0001
30-34	1.00	-	-	-	1.00	-	-	-	1.00	-	-	-
35-39	1.20	1.19	1.21	<.0001	1.08	1.07	1.09	<.0001	1.08	1.07	1.09	<.0001
40-44	1.42	1.39	1.44	<.0001	1.19	1.17	1.21	<.0001	1.18	1.16	1.21	<.0001
45-49	1.48	1.43	1.53	<.0001	1.23	1.19	1.28	<.0001	1.23	1.19	1.27	<.0001
>=50	1.45	1.36	1.54	<.0001	1.24	1.16	1.32	<.0001	1.23	1.15	1.31	<.0001

Model 1: crude model

Model 2: with adjustment for maternal age, sex, parity, delivery method, and birth year

Model 3: with adjustment for maternal age, sex, parity, delivery method, birth year, insurance level, and urbanization

SGA: small for gestational age; LGA: large for gestational age; OR: odds ratio; LCL: lower confidence limit; UCL: upper confidence limit

Supplementary Table 4. Association of paternal age with the risk of perinatal outcomes (adjustment for continuous maternal age)

	Logistic regression											
	Model 1				Model 2				Model 3			
	OR	LCL	UCL	p-value	OR	LCL	UCL	p-value	OR	LCL	UCL	p-value
Under-5 mortality												
Paternal age												
<20	2.72	2.18	3.39	<.0001	2.12	1.64	2.75	<.0001	1.78	1.38	2.31	<.0001
20-24	1.69	1.54	1.84	<.0001	1.30	1.15	1.47	<.0001	1.13	1.00	1.28	0.0511
25-29	1.22	1.14	1.29	<.0001	1.07	1.00	1.15	0.0683	1.02	0.94	1.09	0.6977
30-34	1.00	-	-	-	1.00	-	-	-	1.00	-	-	-
35-39	1.05	0.99	1.12	0.1126	1.16	1.08	1.25	0.0001	1.09	1.01	1.18	0.0244
40-44	1.32	1.20	1.45	<.0001	1.59	1.40	1.80	<.0001	1.31	1.15	1.48	<.0001
45-49	1.34	1.11	1.62	0.0029	1.93	1.54	2.41	<.0001	1.35	1.07	1.69	0.0099
>=50	1.81	1.31	2.49	0.0003	2.72	1.88	3.93	<.0001	1.59	1.10	2.31	0.0148
Under-1 mortality												
Paternal age												
<20	2.68	2.05	3.50	<.0001	2.14	1.56	2.92	<.0001	1.78	1.31	2.44	0.0003
20-24	1.57	1.41	1.76	<.0001	1.26	1.08	1.46	0.0026	1.08	0.94	1.26	0.2850
25-29	1.16	1.08	1.25	0.0001	1.05	0.96	1.14	0.3301	0.99	0.91	1.08	0.8171
30-34	1.00	-	-	-	1.00	-	-	-	1.00	-	-	-
35-39	1.07	0.99	1.16	0.0918	1.15	1.05	1.26	0.0025	1.08	0.99	1.18	0.1027
40-44	1.30	1.16	1.46	<.0001	1.51	1.30	1.75	<.0001	1.24	1.06	1.44	0.0056
45-49	1.32	1.05	1.67	0.0186	1.83	1.41	2.38	<.0001	1.27	0.97	1.66	0.0794
>=50	1.72	1.16	2.55	0.0072	2.44	1.56	3.79	<.0001	1.41	0.90	2.21	0.1343
Premature birth												
Paternal age												
<20	1.62	1.52	1.72	<.0001	1.81	1.69	1.95	<.0001	1.75	1.63	1.88	<.0001
20-24	1.14	1.11	1.16	<.0001	1.22	1.19	1.26	<.0001	1.19	1.15	1.22	<.0001

25-29	0.98	0.97	1.00	0.0163	1.02	1.00	1.04	0.0167	1.01	0.99	1.02	0.4090
30-34	1.00	-	-	-	1.00	-	-	-	1.00	-	-	-
35-39	1.14	1.12	1.15	<.0001	1.07	1.06	1.09	<.0001	1.07	1.05	1.08	<.0001
40-44	1.40	1.38	1.43	<.0001	1.26	1.22	1.29	<.0001	1.22	1.18	1.25	<.0001
45-49	1.62	1.56	1.68	<.0001	1.41	1.34	1.48	<.0001	1.33	1.27	1.40	<.0001
>=50	1.73	1.61	1.85	<.0001	1.48	1.36	1.61	<.0001	1.35	1.24	1.47	<.0001
Low birth weight												
Paternal age												
<20	1.90	1.79	2.01	<.0001	1.62	1.51	1.74	<.0001	1.56	1.45	1.68	<.0001
20-24	1.38	1.35	1.41	<.0001	1.26	1.22	1.30	<.0001	1.22	1.18	1.25	<.0001
25-29	1.06	1.05	1.07	<.0001	1.02	1.00	1.04	0.0444	1.01	0.99	1.02	0.6118
30-34	1.00	-	-	-	1.00	-	-	-	1.00	-	-	-
35-39	1.06	1.04	1.07	<.0001	1.10	1.08	1.12	<.0001	1.09	1.07	1.11	<.0001
40-44	1.26	1.23	1.28	<.0001	1.33	1.29	1.37	<.0001	1.28	1.24	1.32	<.0001
45-49	1.46	1.40	1.53	<.0001	1.58	1.50	1.66	<.0001	1.47	1.39	1.55	<.0001
>=50	1.59	1.47	1.71	<.0001	1.78	1.63	1.96	<.0001	1.60	1.46	1.76	<.0001
Congenital defects												
Paternal age												
<20	1.35	1.07	1.70	0.0103	1.31	1.00	1.72	0.0478	1.27	0.97	1.66	0.0872
20-24	1.05	0.97	1.14	0.2384	1.04	0.94	1.16	0.4361	1.02	0.91	1.13	0.7700
25-29	0.99	0.95	1.04	0.8143	0.99	0.94	1.05	0.7745	0.99	0.93	1.05	0.6089
30-34	1.00	-	-	-	1.00	-	-	-	1.00	-	-	-
35-39	1.08	1.03	1.14	0.0008	1.06	1.00	1.12	0.0506	1.05	0.99	1.11	0.1036
40-44	1.20	1.12	1.29	<.0001	1.12	1.01	1.24	0.0270	1.09	0.98	1.20	0.1002
45-49	1.29	1.11	1.48	0.0007	1.18	0.99	1.41	0.0688	1.12	0.94	1.34	0.2215
>=50	1.32	1.00	1.74	0.0501	1.14	0.83	1.58	0.4163	1.05	0.76	1.45	0.7624
Low five minutes Apgar score												
Paternal age												

<20	1.47	1.13	1.90	0.0036	1.46	1.09	1.95	0.0116	1.37	1.02	1.84	0.0338
20-24	1.20	1.10	1.31	<.0001	1.17	1.05	1.30	0.0044	1.11	1.00	1.24	0.0493
25-29	1.06	1.01	1.12	0.0335	1.03	0.96	1.09	0.4089	1.01	0.95	1.08	0.7607
30-34	1.00	-	-	-	1.00	-	-	-	1.00	-	-	-
35-39	1.10	1.04	1.17	0.0006	1.07	1.01	1.14	0.0357	1.05	0.99	1.12	0.1214
40-44	1.52	1.40	1.64	<.0001	1.34	1.21	1.48	<.0001	1.27	1.15	1.40	<.0001
45-49	1.90	1.65	2.18	<.0001	1.62	1.37	1.92	<.0001	1.47	1.24	1.74	<.0001
>=50	2.58	2.04	3.27	<.0001	1.95	1.48	2.56	<.0001	1.68	1.27	2.21	0.0002
SGA												
Paternal age												
<20	1.84	1.75	1.93	<.0001	1.24	1.17	1.32	<.0001	1.21	1.14	1.28	<.0001
20-24	1.50	1.47	1.52	<.0001	1.18	1.15	1.21	<.0001	1.15	1.13	1.18	<.0001
25-29	1.16	1.15	1.18	<.0001	1.04	1.02	1.05	<.0001	1.03	1.01	1.04	0.0002
30-34	1.00	-	-	-	1.00	-	-	-	1.00	-	-	-
35-39	0.92	0.91	0.94	<.0001	1.05	1.03	1.06	<.0001	1.04	1.02	1.06	<.0001
40-44	0.93	0.91	0.95	<.0001	1.17	1.14	1.20	<.0001	1.13	1.10	1.16	<.0001
45-49	1.02	0.98	1.06	0.4225	1.36	1.29	1.42	<.0001	1.28	1.22	1.34	<.0001
>=50	1.09	1.01	1.17	0.0225	1.55	1.42	1.68	<.0001	1.42	1.30	1.55	<.0001
LGA												
Paternal age												
<20	0.45	0.41	0.49	<.0001	0.73	0.66	0.80	<.0001	0.73	0.66	0.80	<.0001
20-24	0.65	0.63	0.66	<.0001	0.86	0.84	0.88	<.0001	0.86	0.83	0.88	<.0001
25-29	0.83	0.83	0.84	<.0001	0.95	0.93	0.96	<.0001	0.95	0.93	0.96	<.0001
30-34	1.00	-	-	-	1.00	-	-	-	1.00	-	-	-
35-39	1.20	1.19	1.21	<.0001	1.02	1.01	1.03	0.0062	1.02	1.01	1.03	0.0068
40-44	1.42	1.39	1.44	<.0001	1.03	1.00	1.05	0.0348	1.03	1.00	1.05	0.0455
45-49	1.48	1.43	1.53	<.0001	0.95	0.91	0.99	0.0170	0.95	0.91	0.99	0.0120
>=50	1.45	1.36	1.54	<.0001	0.80	0.74	0.87	<.0001	0.80	0.74	0.86	<.0001

Model 1: crude model

Model 2: with adjustment for maternal age, sex, parity, delivery method, and birth year

Model 3: with adjustment for maternal age, sex, parity, delivery method, birth year, insurance level, and urbanization

SGA: small for gestational age; LGA: large for gestational age; OR: odds ratio; LCL: lower confidence limit; UCL: upper confidence limit

Supplementary Table 5. Association of maternal age with the risk of perinatal outcomes

	Logistic regression											
	Model 1				Model 2				Model 3			
	OR	LCL	UCL	p-value	OR	LCL	UCL	p-value	OR	LCL	UCL	p-value
Under-5 mortality												
Maternal age												
<20	2.49	2.12	2.93	<.0001	2.45	1.89	3.18	<.0001	1.66	1.28	2.15	0.0001
20-24	1.63	1.51	1.76	<.0001	1.73	1.48	2.02	<.0001	1.34	1.15	1.57	0.0002
25-29	1.21	1.14	1.29	<.0001	1.28	1.14	1.44	<.0001	1.16	1.03	1.30	0.0137
30-34	1.00	-	-	-	1.00	-	-	-	1.00	-	-	-
35-39	1.10	1.03	1.17	0.0046	1.05	0.91	1.22	0.4856	1.11	0.96	1.29	0.1531
>=40	1.41	1.30	1.54	<.0001	1.23	0.96	1.57	0.0980	1.31	1.02	1.67	0.0313
Under-1 mortality												
Maternal age												
<20	2.34	1.92	2.85	<.0001	2.26	1.65	3.10	<.0001	1.52	1.11	2.08	0.0094
20-24	1.50	1.37	1.66	<.0001	1.58	1.31	1.91	<.0001	1.22	1.02	1.48	0.0341
25-29	1.17	1.09	1.26	<.0001	1.26	1.10	1.44	0.0010	1.13	0.99	1.30	0.0747
30-34	1.00	-	-	-	1.00	-	-	-	1.00	-	-	-
35-39	1.12	1.04	1.21	0.0033	1.11	0.93	1.32	0.2564	1.17	0.98	1.39	0.0788
>=40	1.40	1.26	1.55	<.0001	1.16	0.87	1.54	0.3037	1.23	0.93	1.64	0.1456
Premature birth												
Maternal age												
<20	1.41	1.35	1.48	<.0001	1.33	1.24	1.42	<.0001	1.23	1.15	1.32	<.0001
20-24	1.08	1.06	1.10	<.0001	1.05	1.01	1.09	0.0070	1.00	0.97	1.04	0.8643
25-29	0.97	0.95	0.98	<.0001	0.97	0.94	1.00	0.0177	0.95	0.93	0.98	0.0001
30-34	1.00	-	-	-	1.00	-	-	-	1.00	-	-	-
35-39	1.14	1.13	1.16	<.0001	1.07	1.04	1.11	<.0001	1.09	1.05	1.12	<.0001
>=40	1.45	1.42	1.47	<.0001	1.17	1.11	1.23	<.0001	1.19	1.13	1.25	<.0001

Low birth weight												
Maternal age												
<20	1.74	1.67	1.82	<.0001	1.82	1.70	1.95	<.0001	1.68	1.57	1.80	<.0001
20-24	1.27	1.25	1.30	<.0001	1.28	1.23	1.34	<.0001	1.22	1.17	1.27	<.0001
25-29	1.03	1.02	1.05	<.0001	1.05	1.02	1.08	0.0007	1.03	1.00	1.06	0.0602
30-34	1.00 -	-		-	1.00 -	-		-	1.00 -	-		-
35-39	1.08	1.06	1.09	<.0001	1.07	1.04	1.11	<.0001	1.09	1.05	1.13	<.0001
>=40	1.31	1.28	1.34	<.0001	1.09	1.03	1.16	0.0042	1.11	1.05	1.18	0.0006
Congenital defects												
Maternal age												
<20	1.39	1.19	1.64	<.0001	1.27	1.01	1.60	0.0383	1.19	0.95	1.50	0.1337
20-24	1.07	1.00	1.14	0.0636	1.00	0.88	1.13	0.9440	0.96	0.84	1.09	0.5049
25-29	1.01	0.96	1.06	0.7628	0.97	0.89	1.06	0.4806	0.96	0.88	1.04	0.3090
30-34	1.00 -	-		-	1.00 -	-		-	1.00 -	-		-
35-39	1.08	1.03	1.14	0.0016	1.08	0.97	1.20	0.1722	1.09	0.98	1.21	0.1327
>=40	1.21	1.13	1.30	<.0001	1.16	0.97	1.39	0.1109	1.17	0.98	1.40	0.0925
Low five minutes Apgar score												
Maternal age												
<20	2.02	1.72	2.36	<.0001	1.39	1.13	1.71	0.0022	1.24	1.00	1.52	0.0491
20-24	1.51	1.41	1.62	<.0001	1.14	1.02	1.27	0.0248	1.06	0.94	1.18	0.3442
25-29	1.16	1.10	1.22	<.0001	1.02	0.95	1.11	0.5617	1.00	0.92	1.08	0.9162
30-34	1.00 -	-		-	1.00 -	-		-	1.00 -	-		-
35-39	1.12	1.06	1.19	<.0001	1.23	1.12	1.36	<.0001	1.25	1.13	1.38	<.0001
>=40	1.54	1.43	1.66	<.0001	1.44	1.23	1.68	<.0001	1.46	1.25	1.71	<.0001
SGA												
Maternal age												
<20	1.86	1.80	1.93	<.0001	1.89	1.79	2.00	<.0001	1.78	1.69	1.88	<.0001
20-24	1.44	1.42	1.46	<.0001	1.39	1.35	1.44	<.0001	1.34	1.30	1.38	<.0001

25-29	1.14	1.13	1.15	<.0001	1.10	1.08	1.13	<.0001	1.09	1.06	1.11	<.0001
30-34	1.00	-	-	-	1.00	-	-	-	1.00	-	-	-
35-39	0.94	0.92	0.95	<.0001	1.02	0.99	1.05	0.1761	1.03	1.00	1.06	0.0483
>=40	0.95	0.93	0.97	<.0001	0.99	0.94	1.04	0.5625	1.00	0.95	1.05	0.9551
LGA												
Maternal age												
<20	0.48	0.46	0.51	<.0001	0.47	0.43	0.51	<.0001	0.47	0.43	0.50	<.0001
20-24	0.68	0.66	0.69	<.0001	0.65	0.63	0.68	<.0001	0.65	0.63	0.67	<.0001
25-29	0.85	0.84	0.86	<.0001	0.86	0.84	0.88	<.0001	0.86	0.84	0.88	<.0001
30-34	1.00	-	-	-	1.00	-	-	-	1.00	-	-	-
35-39	1.19	1.17	1.20	<.0001	1.07	1.05	1.10	<.0001	1.07	1.05	1.10	<.0001
>=40	1.40	1.37	1.42	<.0001	1.09	1.04	1.13	<.0001	1.09	1.04	1.13	<.0001

Model 1: crude model

Model 2: with adjustment for paternal age, sex, parity, delivery method, and birth year

Model 3: with adjustment for paternal age, sex, parity, delivery method, birth year, insurance level, and urbanization

SGA: small for gestational age; LGA: large for gestational age; OR: odds ratio; LCL: lower confidence limit; UCL: upper confidence limit

Supplementary Table 6. Association between parity and perinatal outcomes in the sibling-comparison analyses

	OR*	LCL	UCL	p-value
Under-5 mortality				
Paternal age	0.87	0.86	0.89	<.0001
Parity				
1	1	-	-	-
2	0.81	0.76	0.86	<.0001
>=3	0.25	0.22	0.28	<.0001
Under-1 mortality				
Paternal age	0.88	0.87	0.90	<.0001
Parity				
1	1	-	-	-
2	0.77	0.72	0.83	<.0001
>=3	0.25	0.22	0.29	<.0001
Premature birth				
Paternal age	1.05	1.04	1.05	<.0001
Parity				
1	1	-	-	-
2	1.23	1.21	1.25	<.0001
>=3	1.33	1.29	1.38	<.0001
Low birth weight				
Paternal age	0.97	0.96	0.97	<.0001
Parity				
1	1	-	-	-
2	0.81	0.80	0.83	<.0001
>=3	0.77	0.74	0.80	<.0001
Congenital defect				
Paternal age	0.94	0.93	0.95	<.0001
Parity				
1	1	-	-	-
2	0.85	0.81	0.89	<.0001
>=3	0.66	0.58	0.75	<.0001
Low five minutes Apgar score				
Paternal age	0.89	0.87	0.90	<.0001
Parity				
1	1	-	-	-
2	0.65	0.61	0.70	<.0001
>=3	0.37	0.32	0.43	<.0001
SGA				
Paternal age	0.89	0.89	0.90	<.0001
Parity				

1	1	-	-	-
2	0.57	0.56	0.57	<.0001
>=3	0.49	0.47	0.51	<.0001
LGA				
Paternal age	1.10	1.10	1.10	<.0001
Parity				
1	1	-	-	-
2	1.49	1.47	1.51	<.0001
>=3	1.95	1.88	2.01	<.0001

*with adjustment for sex and delivery method

SGA: small for gestational age; LGA: large for gestational age; OR: odds ratio; LCL: lower confidence limit; UCL: upper confidence limit

Supplementary Table 7. Association of paternal age with the risk of perinatal outcomes stratified by categorical maternal age

	Maternal age															
	<24				25-29				30-34				>=35			
	OR*	LCL	UCL	p-value	OR*	LCL	UCL	p-value	OR*	LCL	UCL	p-value	OR*	LCL	UCL	p-value
Under-5 mortality																
Paternal age																
<20	1.18	0.43	3.24	0.7523	NA	NA	NA	NA	NA	NA	NA	NA	NA	NA	NA	NA
20-24	0.91	0.68	1.22	0.5413	0.70	0.38	1.28	0.2420	NA	NA	NA	NA	NA	NA	NA	NA
25-29	0.97	0.74	1.28	0.8198	0.85	0.71	1.02	0.0757	0.82	0.52	1.28	0.3815	0.46	0.06	3.44	0.4452
30-34	1.00	-	-	-	1.00	-	-	-	1.00	-	-	-	1.00	-	-	-
35-39	1.16	0.72	1.88	0.5464	0.80	0.58	1.10	0.1719	0.90	0.71	1.13	0.3465	1.17	0.70	1.96	0.5556
40-44	0.79	0.29	2.16	0.6428	1.79	1.14	2.80	0.0114	0.97	0.61	1.53	0.8964	1.34	0.80	2.25	0.2696
45-49	NA	NA	NA	NA	NA	NA	NA	NA	NA	NA	NA	NA	1.44	0.84	2.48	0.1853
>=50	NA	NA	NA	NA	2.56	0.63	10.40	0.1899	NA	NA	NA	NA	1.64	0.89	3.02	0.1156
Under-1 mortality																
Paternal age																
<20	0.41	0.06	2.94	0.3711	NA	NA	NA	NA	NA	NA	NA	NA	NA	NA	NA	NA
20-24	0.92	0.65	1.29	0.6251	0.66	0.31	1.40	0.2758	NA	NA	NA	NA	NA	NA	NA	NA
25-29	0.83	0.60	1.16	0.2752	0.90	0.73	1.12	0.3452	0.52	0.27	0.98	0.0440	0.61	0.08	4.68	0.6320
30-34	1.00	-	-	-	1.00	-	-	-	1.00	-	-	-	1.00	-	-	-
35-39	1.08	0.61	1.92	0.7968	0.79	0.54	1.16	0.2339	0.84	0.64	1.11	0.2207	1.25	0.69	2.27	0.4630
40-44	1.08	0.39	2.99	0.8899	1.98	1.18	3.32	0.0095	0.90	0.52	1.56	0.7094	1.38	0.76	2.51	0.2929
45-49	NA	NA	NA	NA	NA	NA	NA	NA	NA	NA	NA	NA	1.52	0.81	2.85	0.1901
>=50	NA	NA	NA	NA	1.84	0.26	13.22	0.5468	NA	NA	NA	NA	1.66	0.81	3.38	0.1659
Premature birth																
Paternal age																
<20	1.09	0.82	1.45	0.5374	1.09	0.26	4.58	0.9103	1.63	0.37	7.23	0.5194	NA	NA	NA	NA
20-24	1.02	0.95	1.10	0.5616	1.07	0.95	1.22	0.2777	1.00	0.64	1.56	0.9867	0.28	0.04	2.01	0.2040

25-29	0.95	0.88	1.02	0.1252	1.00	0.96	1.04	0.9217	0.99	0.91	1.08	0.8248	1.06	0.82	1.38	0.6596
30-34	1.00	-	-	-	1.00	-	-	-	1.00	-	-	-	1.00	-	-	-
35-39	1.04	0.91	1.18	0.6034	1.08	1.01	1.16	0.0329	1.04	0.99	1.09	0.1134	0.97	0.88	1.07	0.5622
40-44	1.19	0.95	1.50	0.1254	1.05	0.90	1.22	0.5462	1.20	1.09	1.32	0.0002	1.14	1.03	1.26	0.0101
45-49	1.28	0.83	1.98	0.2574	1.07	0.76	1.52	0.6907	1.02	0.81	1.29	0.8562	1.28	1.15	1.41	<.0001
>=50	1.82	0.94	3.53	0.0771	0.96	0.52	1.77	0.8904	1.39	0.95	2.02	0.0911	1.32	1.17	1.49	<.0001
Low birth weight																
Paternal age																
<20	1.52	1.17	1.97	0.0020	1.25	0.30	5.26	0.7621	2.57	0.58	11.39	0.2130	NA	NA	NA	NA
20-24	1.19	1.10	1.28	<.0001	1.16	1.01	1.32	0.0318	1.38	0.89	2.16	0.1536	0.70	0.17	2.93	0.6300
25-29	1.04	0.96	1.12	0.3413	0.97	0.92	1.01	0.1182	1.03	0.93	1.14	0.6019	1.28	0.97	1.69	0.0790
30-34	1.00	-	-	-	1.00	-	-	-	1.00	-	-	-	1.00	-	-	-
35-39	1.11	0.97	1.28	0.1232	1.02	0.95	1.10	0.5770	1.02	0.97	1.08	0.4940	0.90	0.80	1.01	0.0639
40-44	1.13	0.88	1.44	0.3403	0.94	0.79	1.11	0.4435	1.21	1.09	1.35	0.0006	1.02	0.91	1.14	0.7914
45-49	1.95	1.33	2.87	0.0007	1.48	1.08	2.05	0.0164	0.88	0.66	1.18	0.4004	1.11	0.98	1.25	0.0921
>=50	2.97	1.67	5.29	0.0002	0.92	0.47	1.79	0.7966	1.29	0.83	2.02	0.2576	1.13	0.98	1.29	0.0998
Congenital defects																
Paternal age																
<20	1.60	0.74	3.46	0.2329	NA	NA	NA	NA	NA	NA	NA	NA	NA	NA	NA	NA
20-24	1.02	0.80	1.29	0.9009	0.97	0.63	1.49	0.8871	0.60	0.08	4.25	0.6047	5.05	0.67	37.98	0.1155
25-29	0.90	0.71	1.13	0.3508	0.93	0.81	1.06	0.2718	1.01	0.75	1.36	0.9487	1.58	0.65	3.82	0.3107
30-34	1.00	-	-	-	1.00	-	-	-	1.00	-	-	-	1.00	-	-	-
35-39	0.76	0.47	1.23	0.2663	1.04	0.82	1.30	0.7616	1.06	0.90	1.24	0.4852	1.28	0.87	1.88	0.2084
40-44	1.30	0.66	2.59	0.4493	0.79	0.45	1.38	0.4063	1.05	0.76	1.47	0.7594	1.39	0.95	2.04	0.0938
45-49	1.18	0.29	4.81	0.8174	1.02	0.33	3.18	0.9770	0.82	0.34	1.98	0.6569	1.49	1.00	2.24	0.0532
>=50	NA	NA	NA	NA	0.94	0.13	6.74	0.9515	0.54	0.08	3.87	0.5422	1.50	0.93	2.43	0.0979
Low five minutes Apgar score																
Paternal age																

<20	2.21	1.15	4.25	0.0174	NA	NA	NA	NA	NA	NA	NA	NA	NA	NA	NA	NA
20-24	1.11	0.89	1.39	0.3718	1.08	0.75	1.56	0.6860	0.96	0.24	3.87	0.9521	NA	NA	NA	NA
25-29	1.04	0.84	1.29	0.7121	1.04	0.93	1.18	0.4825	0.95	0.72	1.26	0.7320	0.90	0.39	2.12	0.8170
30-34	1.00	-	-	-	1.00	-	-	-	1.00	-	-	-	1.00	-	-	-
35-39	1.30	0.91	1.85	0.1529	0.99	0.80	1.22	0.9270	0.96	0.83	1.12	0.6202	0.94	0.70	1.27	0.6830
40-44	0.69	0.30	1.57	0.3739	1.27	0.86	1.88	0.2252	1.23	0.93	1.63	0.1444	1.20	0.88	1.62	0.2480
45-49	NA	NA	NA	NA	1.04	0.39	2.80	0.9374	0.77	0.35	1.74	0.5350	1.53	1.11	2.12	0.0105
>=50	5.74	2.04	16.11	0.0009	0.68	0.09	4.85	0.6959	0.41	0.06	2.96	0.3794	1.85	1.25	2.73	0.0020
SGA																
Paternal age																
<20	1.26	1.01	1.56	0.0367	2.94	1.25	6.91	0.0138	0.84	0.11	6.41	0.8690	NA	NA	NA	NA
20-24	1.21	1.14	1.28	<.0001	1.21	1.09	1.34	0.0003	1.67	1.18	2.35	0.0037	1.23	0.48	3.18	0.6646
25-29	1.05	1.00	1.11	0.0733	1.05	1.01	1.08	0.0069	1.13	1.04	1.22	0.0029	1.30	1.02	1.67	0.0330
30-34	1.00	-	-	-	1.00	-	-	-	1.00	-	-	-	1.00	-	-	-
35-39	0.94	0.84	1.05	0.2585	1.02	0.96	1.09	0.4672	0.95	0.91	0.99	0.0262	0.89	0.80	0.98	0.0163
40-44	1.18	0.99	1.42	0.0651	0.94	0.83	1.08	0.3873	1.06	0.97	1.16	0.2298	0.87	0.79	0.96	0.0058
45-49	1.43	1.02	1.99	0.0362	1.15	0.87	1.51	0.3344	0.99	0.79	1.24	0.9328	0.89	0.80	0.98	0.0237
>=50	1.54	0.88	2.70	0.1317	0.84	0.50	1.44	0.5312	0.78	0.50	1.21	0.2670	0.88	0.78	1.00	0.0501
LGA																
Paternal age																
<20	0.59	0.41	0.83	0.0029	0.72	0.17	3.06	0.6589	0.82	0.18	3.65	0.7946	NA	NA	NA	NA
20-24	0.83	0.77	0.89	<.0001	0.76	0.68	0.86	<.0001	0.86	0.60	1.24	0.4174	1.50	0.68	3.29	0.3116
25-29	0.94	0.88	1.01	0.0712	0.89	0.86	0.92	<.0001	0.94	0.88	1.01	0.0917	0.99	0.80	1.23	0.9457
30-34	1.00	-	-	-	1.00	-	-	-	1.00	-	-	-	1.00	-	-	-
35-39	0.91	0.80	1.04	0.1567	1.10	1.04	1.16	0.0011	1.12	1.08	1.16	<.0001	1.04	0.97	1.13	0.2951
40-44	1.25	1.01	1.54	0.0427	1.19	1.06	1.34	0.0034	1.18	1.10	1.27	<.0001	1.22	1.13	1.32	<.0001
45-49	1.08	0.69	1.68	0.7366	1.25	0.96	1.64	0.0949	1.31	1.11	1.55	0.0017	1.31	1.21	1.42	<.0001
>=50	1.66	0.85	3.24	0.1375	0.83	0.49	1.39	0.4672	1.06	0.76	1.47	0.7416	1.33	1.20	1.47	<.0001

*with adjustment for sex, parity, delivery method, birth year, insurance level, and urbanization

SGA: small for gestational age; LGA: large for gestational age; OR: odds ratio; LCL: lower confidence limit; UCL: upper confidence limit

Supplementary Table 8. Association between parity and perinatal outcomes stratified by categorical maternal age in the sibling-comparison analyses

	Maternal age															
	<24				25-29				30-34				>=35			
	OR*	LCL	UCL	p-value	OR*	LCL	UCL	p-value	OR*	LCL	UCL	p-value	OR*	LCL	UCL	p-value
Under-5 mortality																
Paternal age	0.87	0.77	0.98	0.0260	0.78	0.73	0.84	<.0001	0.78	0.73	0.83	<.0001	0.80	0.77	0.84	<.0001
Parity																
1	1.00	-	-	-	1.00	-	-	-	1.00	-	-	-	1.00	-	-	-
2	0.94	0.74	1.18	0.5924	0.71	0.60	0.83	<.0001	0.70	0.60	0.81	<.0001	0.61	0.53	0.71	<.0001
>=3	0.44	0.24	0.80	0.0077	0.19	0.13	0.29	<.0001	0.17	0.12	0.24	<.0001	0.16	0.12	0.22	<.0001
Under-1 mortality																
Paternal age	0.76	0.65	0.89	0.0007	0.76	0.70	0.83	<.0001	0.73	0.68	0.79	<.0001	0.80	0.76	0.84	<.0001
Parity																
1	1.00	-	-	-	1.00	-	-	-	1.00	-	-	-	1.00	-	-	-
2	0.76	0.57	1.02	0.0637	0.66	0.54	0.79	<.0001	0.60	0.51	0.71	<.0001	0.56	0.47	0.67	<.0001
>=3	0.29	0.14	0.63	0.0017	0.17	0.10	0.27	<.0001	0.12	0.08	0.18	<.0001	0.14	0.10	0.20	<.0001
Premature birth																
Paternal age	1.13	1.09	1.18	<.0001	1.11	1.09	1.13	<.0001	1.07	1.06	1.09	<.0001	1.05	1.04	1.06	<.0001
Parity																
1	1.00	-	-	-	1.00	-	-	-	1.00	-	-	-	1.00	-	-	-
2	1.48	1.37	1.60	<.0001	1.39	1.33	1.45	<.0001	1.23	1.19	1.28	<.0001	1.14	1.10	1.19	<.0001
>=3	1.44	1.15	1.80	0.0013	1.67	1.46	1.90	<.0001	1.41	1.26	1.57	<.0001	1.37	1.25	1.49	<.0001
Low birth weight																
Paternal age	0.94	0.90	0.98	0.0023	0.91	0.89	0.93	<.0001	0.90	0.88	0.91	<.0001	0.96	0.95	0.97	<.0001
Parity																
1	1.00	-	-	-	1.00	-	-	-	1.00	-	-	-	1.00	-	-	-
2	0.98	0.91	1.06	0.6509	0.84	0.80	0.88	<.0001	0.77	0.74	0.80	<.0001	0.76	0.73	0.80	<.0001
>=3	0.77	0.60	0.98	0.0328	0.83	0.72	0.96	0.0105	0.68	0.60	0.77	<.0001	0.75	0.68	0.84	<.0001

Congenital defect																
Paternal age	1.04	0.91	1.19	0.6035	0.98	0.91	1.04	0.4720	0.92	0.87	0.97	0.0015	0.97	0.93	1.00	0.0733
Parity																
1	1.00	-	-	-	1.00	-	-	-	1.00	-	-	-	1.00	-	-	-
2	1.19	0.92	1.56	0.1886	0.96	0.83	1.12	0.6131	0.83	0.73	0.95	0.0048	0.81	0.71	0.93	0.0028
>=3	0.62	0.25	1.55	0.3081	1.12	0.71	1.78	0.6239	0.54	0.38	0.76	0.0005	0.69	0.50	0.95	0.0212
Low five minutes Apgar score																
Paternal age	0.90	0.78	1.04	0.1648	0.87	0.80	0.95	0.0019	0.90	0.85	0.97	0.0025	0.91	0.87	0.96	<.0001
Parity																
1	1.00	-	-	-	1.00	-	-	-	1.00	-	-	-	1.00	-	-	-
2	1.00	0.77	1.29	0.9732	0.79	0.66	0.95	0.0106	0.77	0.66	0.90	0.0011	0.63	0.54	0.75	<.0001
>=3	0.41	0.18	0.95	0.0377	0.52	0.29	0.94	0.0293	0.62	0.41	0.95	0.0271	0.46	0.32	0.67	<.0001
SGA																
Paternal age	0.82	0.79	0.85	<.0001	0.81	0.80	0.83	<.0001	0.78	0.77	0.79	<.0001	0.88	0.87	0.89	<.0001
Parity																
1	1.00	-	-	-	1.00	-	-	-	1.00	-	-	-	1.00	-	-	-
2	0.64	0.60	0.68	<.0001	0.60	0.58	0.63	<.0001	0.51	0.49	0.53	<.0001	0.54	0.52	0.56	<.0001
>=3	0.66	0.53	0.82	0.0002	0.48	0.42	0.55	<.0001	0.40	0.35	0.44	<.0001	0.47	0.43	0.52	<.0001
LGA																
Paternal age	1.18	1.13	1.24	<.0001	1.16	1.14	1.18	<.0001	1.19	1.18	1.21	<.0001	1.11	1.10	1.13	<.0001
Parity																
1	1.00	-	-	-	1.00	-	-	-	1.00	-	-	-	1.00	-	-	-
2	1.37	1.25	1.49	<.0001	1.39	1.33	1.45	<.0001	1.57	1.52	1.62	<.0001	1.61	1.55	1.67	<.0001
>=3	1.53	1.14	2.04	0.0041	1.83	1.60	2.10	<.0001	2.25	2.03	2.49	<.0001	2.04	1.88	2.21	<.0001

*with adjustment for sex and delivery method; SGA: small for gestational age; LGA: large for gestational age; OR: odds ratio; LCL: lower confidence limit; UCL: upper confidence limit
